# CoDysAn: A Telemedicine Tool to Improve Awareness and Diagnosis for Patients With Congenital Dyserythropoietic Anemia

**DOI:** 10.3389/fphys.2019.01063

**Published:** 2019-09-13

**Authors:** Cristian Tornador, Edgar Sánchez-Prados, Beatriz Cadenas, Roberta Russo, Veronica Venturi, Immacolata Andolfo, Ines Hernández-Rodriguez, Achille Iolascon, Mayka Sánchez

**Affiliations:** ^1^BloodGenetics S.L., Barcelona, Spain; ^2^Teresa Moreto Foundation, Barcelona, Spain; ^3^Bioinformatics for Health Sciences Master Programme, Universitat Pompeu Fabra, Barcelona, Spain; ^4^Whole Genix SL., Barcelona, Spain; ^5^Universitat de Vic-Universitat Central de Catalunya, Vic, Spain; ^6^Iron Metabolism: Regulation and Diseases Group, Josep Carreras Leukaemia Research Institute, Campus Can Ruti, Barcelona, Spain; ^7^Department of Molecular Medicine and Medical Biotechnologies, University of Naples Federico II, Naples, Italy; ^8^CEINGE–Biotecnologie Avanzate, Naples, Italy; ^9^Iron Metabolism: Regulation and Diseases Group, Department of Basic Sciences, Faculty of Medicine and Health Sciences, Universitat Internacional de Catalunya, Barcelona, Spain; ^10^Haematology Service, Hospital Germans Trias i Pujol University Hospital, Oncology Catalan Institute, Barcelona, Spain

**Keywords:** telemedicine tool, congenital dyserythropoietic anemia, diagnosis, algorithm, hematological disease

## Abstract

Congenital Dyserythropoietic Anemia (CDA) is a heterogeneous group of hematological disorders characterized by chronic hyporegenerative anemia and distinct morphological abnormalities of erythroid precursors in the bone marrow. In many cases, a final diagnosis is not achieved due to different levels of awareness for the diagnosis of CDAs and lack of use of advanced diagnostic procedures. Researchers have identified five major types of CDA: types I, II, III, IV, and X-linked dyserythropoietic anemia and thrombocytopenia (XLDAT). Proper management in CDA is still unsatisfactory, as the different subtypes of CDA have different genetic causes and different but overlapping patterns of signs and symptoms. For this reason, we developed a new telemedicine tool that will help doctors to achieve a faster diagnostic for this disease. Using open access code, we have created a responsive webpage named CoDysAn (**Co**ngenital **Dys**erythropoietic **An**emia) that includes practical information for CDA awareness and a step-by-step diagnostic tool based on a CDA algorithm. The site is currently available in four languages (Catalan, Spanish, Italian, and English). This telemedicine webpage is available at http://www.codysan.eu.

## Introduction

Congenital Dyserythropoietic Anemia (CDA) is a heterogeneous group of hematological disorders characterized by chronic hyporegenerative anemia and distinct morphological abnormalities of erythroid precursors in the bone marrow. Patients with CDA present congenital and chronic anemia of variable degree with a reticulocytosis not corresponding to the degree of anemia (ineffective erythropoiesis), jaundice and frequently splenomegaly and/or hepatomegaly (Iolascon et al., 2012, 2013).

Five classical types of CDAs (I–II–III–IV and XLTDA) have been defined based on bone marrow morphology. Among all types, CDA type II is the most common and well-known form. Genetically, CDA type Ia (OMIN 224120) and CDA type Ib (OMIM 615631) are caused by mutations in codanin 1 (*CDAN1*) (chr 15q15.2) and *C15orf41* (chr15q14) genes, respectively (Dgany et al., 2002; Babbs et al., 2013). CDA type II (OMIM 224100) is due to pathogenic variants in Sec23 homolog B, coat complex II component (*SEC23B*) gene (chr20p11.23) (Bianchi et al., 2009; Schwarz et al., 2009). Few patients with CDA type III (OMIM 105600) have been described: they present the same mutation in the Kinesin Family Member 23 (*KIF23*) gene (chr15q23) (Liljeholm et al., 2013). CDA type IV (OMIM 613673) is due to mutations in the Kruppel Like Factor 1 (*KLF1*) gene (chr19p13.13) (Arnaud et al., 2010; Jaffray et al., 2013). Finally, X-linked dyserythropoietic anemia and thrombocytopenia (XLDAT) (OMIM 300367) is caused by mutations in transcription factor GATA Binding Protein 1 (*GATA1*) gene (chr Xp11.23) (Nichols et al., 2000; Del Vecchio et al., 2005). CDA types I and II are inherited in an autosomal recessive manner, CDA type III and IV present an autosomal dominant inheritance pattern and X-linked dyserythropoietic anemia with thrombocytopenia has an X-linked mode of inheritance.

Depending on the type of CDA, different treatments have been established. Allogenic bone marrow transplantation has been successfully employed in a few severe cases of CDAI and CDAII. CDA III patients may require a transfusion only during times of extreme anemia e.g., pregnancy or surgery. Treatment focuses on hemoglobin normalization with the administration of interferon (IFN) alpha is used with success in CDA I patients with *CDAN1* mutations; however, patients bearing a mutation in a different gene i.e., *C15ORF41* were unresponsive to this same treatment. Severe cases of fetal anemia associated with CDAI, CDAII, and XLTDA may require intrauterine transfusions. Blood iron levels should be closely monitored in CDA I, CDAII, and other CDA patients undergoing regular transfusions. In these cases, morbidity may be severe due to iron overload complications that can be fatal if left untreated (Gambale et al., 2016; Palmer et al., 2018); therefore, it is imperative to monitor iron overload and induce iron depletion, when needed, by iron chelation. This working classification of CDA is still in use in clinical practice; however, the identification of the mutated genes involved in the majority of CDA subgroups will improve the diagnostic possibilities and allow a better classification of CDA patients. At present, in many cases, a final diagnosis is not achieved due to different levels of awareness for the diagnosis of CDAs and lack of use of advanced diagnostic procedures. In addition, there are families that fulfill the general definition of CDAs, but do not conform to any of the classical CDA variants. Therefore, it is very plausible that new forms of CDA may exist. These new forms will be possibly identified if a proper diagnosed is achieved in each patient suspected with CDA. Toward this goal, we have developed a new telemedicine tool named CoDysAn (**Co**ngenital **Dys**erythropoietic **An**emia) for the management and diagnosis of patients with this disease.

The aim of CoDysAn webpage is to provide a freely accessible website where general public, patients and medical doctors can better understand and learn more about this disease. Moreover, CoDysAn web page includes a diagnosis algorithm tool to ease the classification and diagnostic of CDA types.

## Methods

### Patients and Validation

CoDysAn web algorithm has been developed with a set of 24 patients genetically diagnosed of different types of CDA (18 CDA type II, 1 CDA type Ib, 4 CDA type Ia, and 1 XLTDA) and with a set of 19 additional patients genetically diagnosed of non-CDA hereditable anemias including eight hereditary spherocytosis, four patients with pyruvate kinase defects, one patient with pyruvate kinase defect and a beta thalassemia trait, one patient with defects in hemolytic anemia due to adenylate kinase deficiency (AK1) gene, one patient with X-linked sideroblastic anemia, three patients with dehydrated hereditary stomatocytosis type 1 (DHS1) and one patient with dehydrated hereditary stomatocytosis type 2 (DHS2). A different set of 23 CDAII patients was utilized to independently validate the algorithm. Patients were previously reported (Iolascon et al., 2009; Schwarz et al., 2009; Russo et al., 2010, 2011, 2013, 2014, 2016, 2018; Unal et al., 2014; Andolfo et al., 2015, 2018; Di Pierro et al., 2015) and diagnosed at the Medical Genetics Unit of A.O.U. Federico II, CEINGE–Biotecnologie Avanzate (Napoli).

### Design of Web Server

CoDysAn is implemented in PHP, HTML5, CSS, and Javascript. The web server is executed in a XAMPP. Network visualization and interactive exploration modules are based on several open-source projects: Bootstrap, jQuery and Filezilla. The source code of the diagnostic tool algorithm is implemented in php at http://www.codysan.eu/diagnostics-tool.html. It is integrated within this web page between lines 661 and 1,204 in four steps corresponding to the four steps of the form. The code can be checked by typing in a browser: “view-source: http://www.codysan.eu/diagnostics-tool.html.”

### Implementation

CoDysAn algorithm is based on the diagnostic workflow previously proposed (Iolascon et al., 2012; Gambale et al., 2016). This algorithm is based on hematological parameters depending on age and gender (Table 1). Age is split in three groups: from 0 to 6 months old; from 6 months to 12 years old; and older than 12 years. Hematological tested parameters include: hemoglobin levels, mean corpuscular volume (MCV), reticulocytes count and platelets count. Exclusion of other possible causes of anemia is also considered in the final step of the algorithm. References values for hematological data are adapted from general hematological reference books (Rabinovitch, 1990; Wakeman et al., 2007; Hoffman et al., 2018).

**Table 1:** Parameter thresholds used by the diagnostic CoDysAn algorithm.

**Parameter**		**0–6 months**	**6 months to 12 years**	**>12 years**	**Units**
Hemoglobin	M	9.5–18	11–15.5	13–17.5	g/dL
	F	9.5–18	11–15.5	12–16	
MCV^*^	M	77.5–111.5	74–89.5	80–100	fL
	F	77.5–111.5	74–89.5	80–100	
Reticulocytes	M	61–134	24–114	29–95	× 10^9^/L
	F	67–142	40–162	27–91	
Platelets	M	145–450	145–450	145–450	× 10^9^/L
	F	145–450	145–450	145–450	

* *MCV stands for Mean Corpuscular Volume. M stands for male and F stands for female*.

## Results

### CoDySan Scope

**Figure 1:**
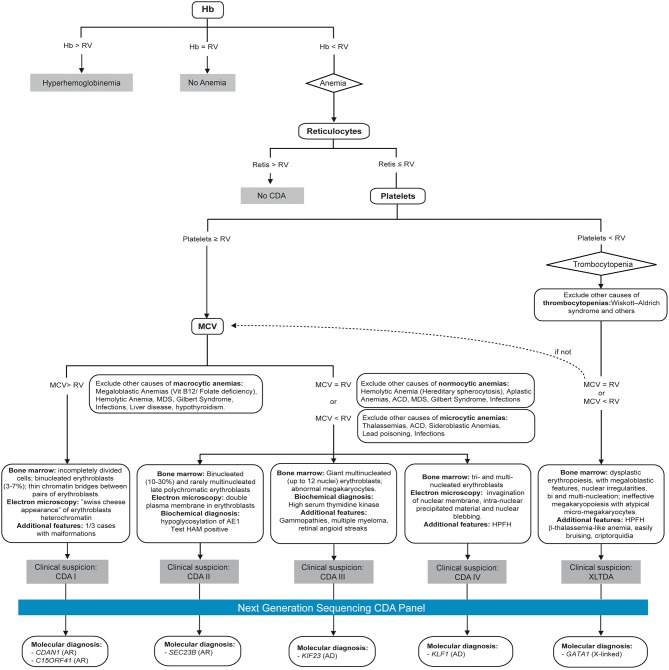
Diagnostic algorithm used by the CoDysAn telemedicine tool. Hb, hemoglobin; RV, reference value; MCV, mean corpuscular volume; HPFH, hereditary persistence of fetal hemoglobin; AR, autosomal recessive; AD, autosomal dominant.

Following previous experience of the group in developing telemedicine tools for management and diagnosis of patients (HIGHFERRITIN Web server http://highferritin.imppc.org/ tool) (Altes et al., 2014), we have developed CoDySan web tool. CoDySan is a user-friendly webpage for a better awareness on the rare hereditary hematological diseases, congenital dyserythropoietic anemias (CDAs). CoDySan webpage includes a step-by-step diagnostic algorithm based on Figure 1. The site is freely available at URL http://www.codysan.eu in four languages (Catalan, Spanish, Italian, and English).

### Webpage Structure and Design

The CoDysAn website is currently containing seven sections (see Supplementary Figure 1 to visualize several screenshots of different sections of CoDysAn webpage): A home or main webpage section including links to other sections of the site to make information more accessible; The CoDysAn section, where one can found information about congenital dyserythropoietic anemia (CDA) disease, the CoDysAn project as a whole research project, the privacy policy, cookies policy and medical disclaimer reminding that, as any other telemedicine project, CoDysAn diagnostic tool is a preliminary diagnostic test and expert medical doctors should be contacted for a conclusive diagnosis; The diagnostic section, including the CDA algorithm flowchart (Figure 1) and a step-by-step diagnostic tool with specific instructions on how to use it; The collaborators section, including links to the contributors for the CoDysAn project, patient associations and links to similar web tools, such as HIGHFERRITIN web server; A resource section, including news on the CoDysAn project, bibliographical references and reference values used for the diagnostic algorithm (see also Table 1); An opinion section containing a Google form that allows users to express their opinion and degree of satisfaction with the website; A contact section, where users can directly contact CoDysAn developers to address any doubt regarding the webpage.

### Diagnostic Telemedicine Tool

The diagnostic algorithm used for setting up the CoDysAn diagnostic tool is depicted in Figure 1. A step-by-step and user-friendly form will progressively ask relevant patient information; in the first stage, age, gender and hemoglobin levels should be provided to discern if the patient has hyperhemoglobinemia (high hemoglobin values in regards to the reference value), anemia or if the values are inside the normal range, in which case the web tool will return a text indicating that there is no anemia.

Due to fluctuations in the hematological parameters, the algorithm correlates to the reference values for the hematological provided data (hemoglobin level, reticulocytes, platelets, etc.) according to age and gender, see Table 1. To simplify the algorithm, we have only considered three different age ranges, from 0 to 6 months, from 6 to 12 years, and older than 12 years old.

If anemia is detected, i.e., the hemoglobin levels are below the normal values for the indicated gender and age range, the algorithm will ask for three additional hematological parameters: mean corpuscular volume (MCV), reticulocytes count, and platelets count.

Users can change the input units of the provided hematological parameter. These values are converted to the international system of reference units and the value is used to check if the parameters are within range for their given thresholds (see Table 1).

Depending on the data provided, a new form will appear asking to exclude specific possible causes of macrocytic, normocytic or microcytic anemia. At least one alternative cause of anemia should be excluded to proceed with the diagnostic tool. In the following step, the user is asked to select additional patient's clinical or biochemical factors, such as binucleated erythroblasts, malformations or electron microscopy features.

Finally, depending on the provided information, CoDySan tool will return a result of clinical suspicion (any of the CDA types) if it applies, or a brief explanation if there is no clinical suspicion of CDA.

If a clinical suspicion of CDA is indicated, the user has the option to search for world-wide genetic laboratories that provide clinical diagnostic tests for a particular CDA gene via the button “Search Lab.” The list of world-wide genetic laboratories is taken from the NCBI's Genetic Testing Registry (GTR) webpage (Rubinstein et al., 2013). There is also the possibility to refresh the webpage and perform a new diagnostic test via the button “New diagnostic.”

### Validation

The CodysAn algorithm has been designed with 43 patients with hereditable anemia, including 24 patients genetically diagnosed with different types of CDA (18 CDA type II, 1 CDA type Ib, 4 CDA type IIa, and 1 XLTDA) and 19 additional patients genetically diagnosed with non-CDA hereditable anemias. The algorithm achieved a specificity of 89.5% and a sensibility of 87.5%. An additional set of 23 patients (all CDA II) was utilized to validate the algorithm, which returned a specificity of 87%.

## Discussion

Telemedicine webpages and tools are significantly changing the way medical doctors and patients approach health care and diagnosis (Dinesen et al., 2016). CoDysAn telemedicine tool is a webpage intended to increase awareness about the rare disease CDA as, currently, patients suffering from this disease are under-diagnosed (Russo et al., 2014). The content of the webpage serves as an informative and training resource for the general public, patients and medical doctors. The use of this tool presents limits: patients should be considered as a whole entity and multiple biochemical determinations are needed due to daily parameters' variability within the same subject. Although hematological reference ranges are useful in results interpretation and in clinical decision-making, it should be borne in mind that variations within the population may affect some outcomes. CoDysAn incorporates a diagnostic algorithm that proved to be useful for a preliminary diagnostic. It will help medical doctors to know which molecular diagnostics they should request, reducing time and effort necessary for the diagnostic of CDA and allowing a direct implementation of a proper treatment once reached a definitive molecular diagnosis. Few reference centers are now offering genetic diagnostic panels screening the six known genes causing CDA. CoDySan algorithm is connected to the NCBI Genetic Testing Registry (GTR) in a way to inform medical doctors about the existence of these accredited diagnostic centers to perform a complete genetic test, if required. This telemedicine tool aims to inform the general public and aid in the diagnosis of CDA. It is not intended as an attempt to practice medicine or provide specific medical advice and it should not be used to replace or overrule a qualified health care provider's judgment. Users should not rely upon this website for self-medication. We believe that CoDysAn webpage will positively contribute to improve medical and scientific communication on the anemia field.

## Data Availability

All datasets generated for this study are included in the manuscript and/or the Supplementary Files.

## Author Contributions

MS designed the webpage, designed the study, and wrote the manuscript. ES-P, CT, and BC created the webpage and wrote the diagnostic algorithm. BC designed Figure 1. IA and RR translated the CoDysAn webpage to Italian. AI, IA, and RR provided the patients data to test CoDysAn algorithm. VV wrote and revised the manuscript. IH-R helped with reference values and Table 1. All authors read and approved the final version of the manuscript.

### Conflict of Interest Statement

CT was employed by company BloodGenetics SL. BC was employed by company Whole Genix SL. The remaining authors declare that the research was conducted in the absence of any commercial or financial relationships that could be construed as a potential conflict of interest.

## References

[B1] Altes A. Perez-Lucena M. J. Bruguera M. Grp Iberico F. (2014). Systematic approach to the diagnosis of hyperferritinemia. Med. Clin. (Barc). 142, 412–417. 10.1016/j.medcli.2013.06.010 24018249

[B2] Andolfo I. Russo R. Manna F. Shmukler B. E. Gambale A. Vitiello G.. (2015). Novel Gardos channel mutations linked to dehydrated hereditary stomatocytosis (xerocytosis). Am. J. Hematol. 90, 921–926. 10.1002/ajh.24117 26178367

[B3] Andolfo I. Russo R. Rosato B. E. Manna F. Gambale A. Brugnara C.. (2018). Genotype-phenotype correlation and risk stratification in a cohort of 123 hereditary stomatocytosis patients. Am. J. Hematol. 93, 1509–1517. 10.1002/ajh.25276 30187933

[B4] Arnaud L. Saison C. Helias V. Lucien N. Steschenko D. Giarratana M. C.. (2010). A Dominant mutation in the gene encoding the erythroid transcription factor KLF1 causes a congenital dyserythropoietic anemia. Am. J. Hum. Genet. 87, 721–727. 10.1016/j.ajhg.2010.10.010 21055716 PMC2978953

[B5] Babbs C. Roberts N. A. Sanchez-Pulido L. McGowan S. J. Ahmed M. R. Brown J. M.. (2013). Homozygous mutations in a predicted endonuclease are a novel cause of congenital dyserythropoietic anemia type I. Haematologica 98, 1383–1387. 10.3324/haematol.2013.089490 23716552 PMC3762094

[B6] Bianchi P. Fermo E. Vercellati C. Boschetti C. Barcellini W. Iurlo A.. (2009). Congenital dyserythropoietic anemia type II (CDAII) is caused by mutations in the SEC23B gene. Hum Mutat. 30, 1292–1298. 10.1002/humu.21077 19621418

[B7] Del Vecchio G. C. Giordani L. De Santis A. De Mattia D. (2005). Dyserythropoietic anemia and thrombocytopenia due to a novel mutation in GATA-1. Acta Haematol. 114, 113–116. 10.1159/000086586 16103636

[B8] Dgany O. Avidan N. Delaunay J. Krasnov T. Shalmon L. Shalev H.. (2002). Congenital dyserythropoietic anemia type I is caused by mutations in codanin-1. Am. J. Hum. Genet. 71, 1467–1474. 10.1086/344781 12434312 PMC378595

[B9] Di Pierro E. Russo R. Karakas Z. Brancaleoni V. Gambale A. Kurt I.. (2015). Congenital erythropoietic porphyria linked to GATA1-R216W mutation: challenges for diagnosis. Eur. J. Haematol. 94, 491–497. 10.1111/ejh.12452 25251786

[B10] Dinesen B. Nonnecke B. Lindeman D. Toft E. Kidholm K. Jethwani K.. (2016). Personalized telehealth in the future: a global research agenda. J. Med. Internet Res. 18:e53. 10.2196/jmir.5257 26932229 PMC4795318

[B11] Gambale A. Iolascon A. Andolfo I. Russo R. (2016). Diagnosis and management of congenital dyserythropoietic anemias. Expert Rev. Hematol. 9, 283–296. 10.1586/17474086.2016.1131608 26653117

[B12] Hoffman R. Benz E. J. Jr. Silberstein L. E. Heslop H. Weitz J. Anastasi J. (2018). Hematology: Basic Principles and Practice, 7th Edn. Philadelphia, PA: Elsevier Saunders.

[B13] Iolascon A. Esposito M. R. Russo R. (2012). Clinical aspects and pathogenesis of congenital dyserythropoietic anemias: from morphology to molecular approach. Haematologica 97, 1786–1794. 10.3324/haematol.2012.072207 23065504 PMC3590084

[B14] Iolascon A. Heimpel H. Wahlin A. Tamary H. (2013). Congenital dyserythropoietic anemias: molecular insights and diagnostic approach. Blood 122, 2162–2166. 10.1182/blood-2013-05-468223 23940284 PMC3785118

[B15] Iolascon A. Russo R. Esposito M. R. Asci R. Piscopo C. Perrotta S.. (2009). Molecular analysis of 42 patients with congenital dyserythropoietic anemia type II: new mutations in the SEC23B gene and a search for a genotype-phenotype relationship. Haematologica 95, 708–715. 10.3324/haematol.2009.014985 20015893 PMC2864375

[B16] Jaffray J. A. Mitchell W. B. Gnanapragasam M. N. Seshan S. V. Guo X. Westhoff C. M.. (2013). Erythroid transcription factor EKLF/KLF1 mutation causing congenital dyserythropoietic anemia type IV in a patient of Taiwanese origin: review of all reported cases and development of a clinical diagnostic paradigm. Blood Cells Mol. Dis. 51, 71–75. 10.1016/j.bcmd.2013.02.006 23522491 PMC4560093

[B17] Liljeholm M. Irvine A. F. Vikberg A. L. Norberg A. Month S. Sandström H.. (2013). Congenital dyserythropoietic anemia type III (CDA III) is caused by a mutation in kinesin family member, KIF23. Blood 121, 4791–4799. 10.1182/blood-2012-10-461392 23570799

[B18] Nichols K. E. Crispino J. D. Poncz M. White J. G. Orkin S. H. Maris J. M.. (2000). Familial dyserythropoietic anaemia and thrombocytopenia due to an inherited mutation in GATA1. Nat. Genet. 24, 266–270. 10.1038/73480 10700180 PMC10576470

[B19] Palmer W. C. Vishnu P. Sanchez W. Aqel B. Riegert-Johnson D. Seaman L. A. K.. (2018). Diagnosis and management of genetic iron overload disorders. J. Gen. Intern. Med. 33, 2230–2236. 10.1007/s11606-018-4669-2 30225768 PMC6258594

[B20] Rabinovitch A. (1990). Hematology reference ranges. Arch. Pathol. Lab. Med. 114:1189. 2252409

[B21] Rubinstein W. S. Maglott D. R. Lee J. M. Kattman B. L. Malheiro A. J. Ovetsky M.. (2013). The NIH genetic testing registry: a new, centralized database of genetic tests to enable access to comprehensive information and improve transparency. Nucleic Acids Res. 41, D925–D935. 10.1093/nar/gks1173 23193275 PMC3531155

[B22] Russo R. Andolfo I. Manna F. De Rosa G. De Falco L. Gambale A.. (2016). Increased levels of ERFE-encoding FAM132B in patients with congenital dyserythropoietic anemia type II. Blood 128, 1899–1902. 10.1182/blood-2016-06-724328 27540014

[B23] Russo R. Andolfo I. Manna F. Gambale A. Marra R. Rosato B. E.. (2018). Multi-gene panel testing improves diagnosis and management of patients with hereditary anemias. Am. J. Hematol. 93, 672–682. 10.1002/ajh.25058 29396846

[B24] Russo R. Esposito M. R. Asci R. Gambale A. Perrotta S. Ramenghi U.. (2010). Mutational spectrum in congenital dyserythropoietic anemia type II: identification of 19 novel variants in SEC23B gene. Am. J. Hematol. 85, 915–920. 10.1002/ajh.21866 20941788 PMC3015065

[B25] Russo R. Gambale A. Esposito M. R. Serra M. L. Troiano A. De Maggio I.. (2011). Two founder mutations in the SEC23B gene account for the relatively high frequency of CDA II in the Italian population. Am. J. Hematol. 86, 727–732. 10.1002/ajh.22096 21850656 PMC3258542

[B26] Russo R. Gambale A. Langella C. Andolfo I. Unal S. Iolascon A. (2014). Retrospective cohort study of 205 cases with congenital dyserythropoietic anemia type II: definition of clinical and molecular spectrum and identification of new diagnostic scores. Am. J. Hematol. 89, E169–E175. 10.1002/ajh.23800 25044164

[B27] Russo R. Langella C. Esposito M. R. Gambale A. Vitiello F. Vallefuoco F.. (2013). Hypomorphic mutations of SEC23B gene account for mild phenotypes of congenital dyserythropoietic anemia type II. Blood Cells Mol. Dis. 51, 17–21. 10.1016/j.bcmd.2013.02.003 23453696 PMC3651933

[B28] Schwarz K. Iolascon A. Verissimo F. Trede N. S. Horsley W. Chen W.. (2009). Mutations affecting the secretory COPII coat component SEC23B cause congenital dyserythropoietic anemia type II. Nat. Genet. 41, 936–940. 10.1038/ng.405 19561605

[B29] Unal S. Russo R. Gumruk F. Kuskonmaz B. Cetin M. Sayli T.. (2014). Successful hematopoietic stem cell transplantation in a patient with congenital dyserythropoietic anemia type II. Pediatr. Transplant. 18, E130–E133. 10.1111/petr.12254 24724984

[B30] Wakeman L. Al-Ismail S. Benton A. Beddall A. Gibbs A. Hartnell S.. (2007). Robust, routine haematology reference ranges for healthy adults. Int. J. Lab. Hematol. 29, 279–283. 10.1111/j.1365-2257.2006.00883.x 17617078

