# Supplementary material for: CoDysAn: A Telemedicine Tool to Improve Awareness and Diagnosis for Patients With Congenital Dyserythropoietic Anemia
Source: Front Physiol. 2019 Sep 13;10:1063. doi: 10.3389/fphys.2019.01063 (PMC6753183; doi:10.3389/fphys.2019.01063)
Supplement: Supplementary file 1 [file Table_1.DOCX]

**Supplementary Figure 1. Screenshots of different sections of CoDysAn webpage.** A. CoDysAn home page section. B. CoDysAn section, about the disease webpage. C. Diagnostic section, showing the first step where the user is asked to provide gender, age and hemoglobin level. D. Opinion section, showing a Google form that allows the users to express their opinion and degree of satisfaction with the website.
