# Supplementary figures and images for: CoDysAn: A Telemedicine Tool to Improve Awareness and Diagnosis for Patients With Congenital Dyserythropoietic Anemia
Source: Front Physiol. 2019 Sep 13;10:1063. doi: 10.3389/fphys.2019.01063 (PMC6753183; doi:10.3389/fphys.2019.01063)

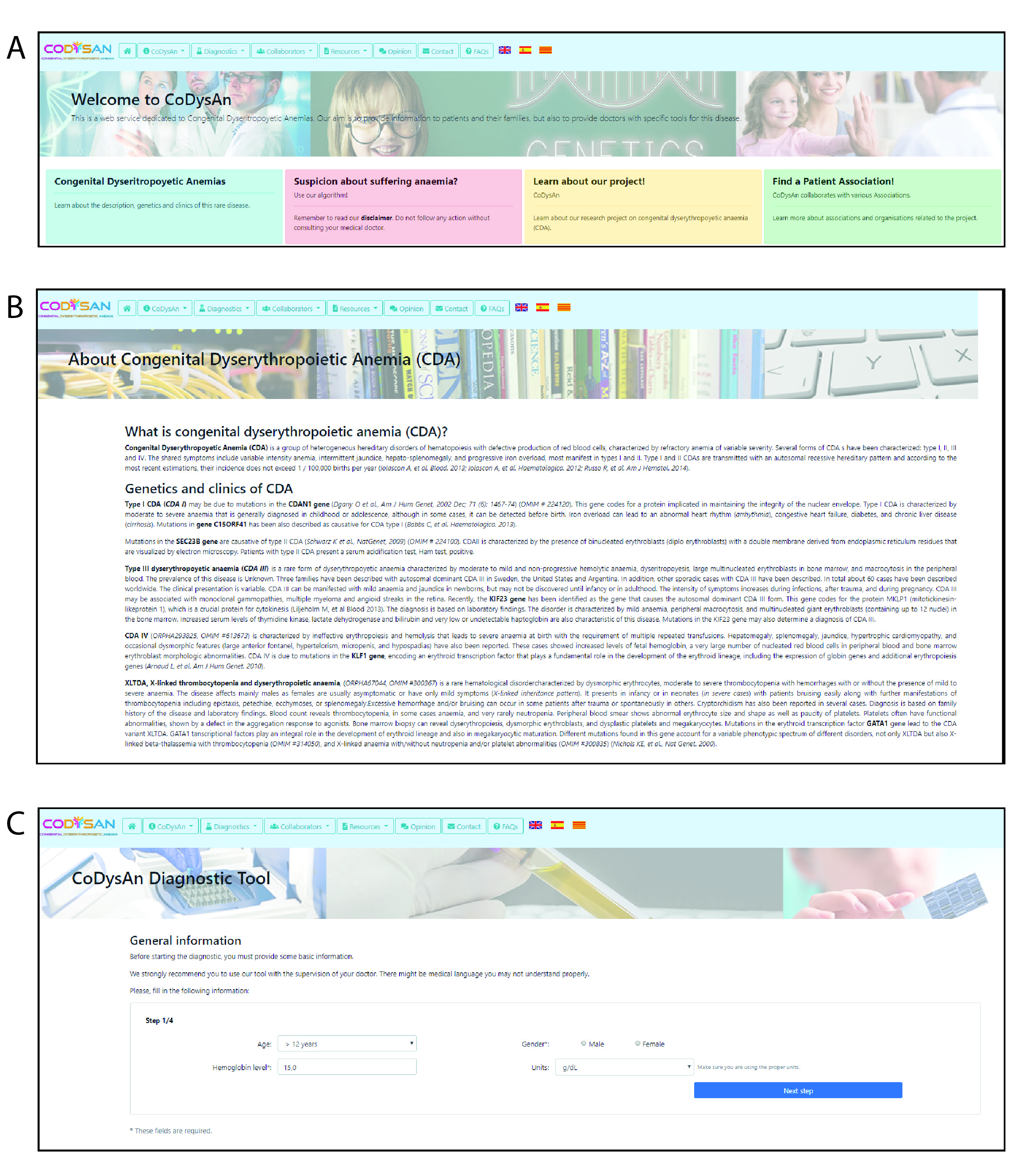

Supplement: Supplementary file 2 [file Image_1.JPEG]

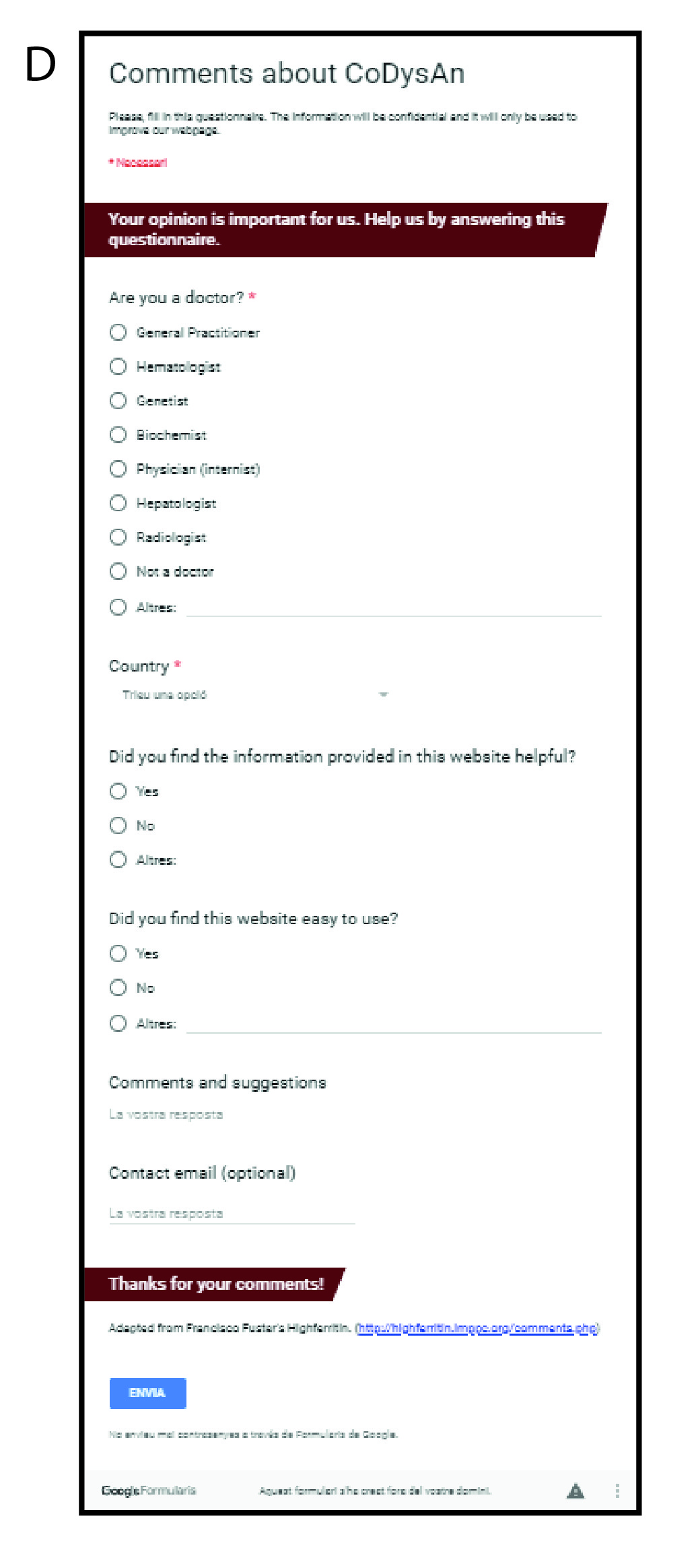

Supplement: Supplementary file 3 [file Image_2.JPEG]
